# Mouse Microbiota Models: Comparing Germ-Free Mice and Antibiotics Treatment as Tools for Modifying Gut Bacteria

**DOI:** 10.3389/fphys.2018.01534

**Published:** 2018-10-31

**Authors:** Elizabeth A. Kennedy, Katherine Y. King, Megan T. Baldridge

**Affiliations:** ^1^Division of Infectious Diseases, Department of Medicine, Center for Genome Sciences and Systems Biology, Washington University School of Medicine, St. Louis, MO, United States; ^2^Section of Infectious Diseases, Department of Pediatrics, Baylor College of Medicine, Houston, TX, United States

**Keywords:** antibiotics, microbiota, gnotobiotic, microbiome, immunity

## Abstract

As the intestinal microbiota has become better appreciated as necessary for maintenance of physiologic homeostasis and also as a modulator of disease processes, there has been a corresponding increase in manipulation of the microbiota in mouse models. While germ-free mouse models are generally considered to be the gold standard for studies of the microbiota, many investigators turn to antibiotics treatment models as a rapid, inexpensive, and accessible alternative. Here we describe and compare these two approaches, detailing advantages and disadvantages to both. Further, we detail what is known about the effects of antibiotics treatment on cell populations, cytokines, and organs, and clarify how this compares to germ-free models. Finally, we briefly describe recent findings regarding microbiota regulation of infectious diseases and other immunologic challenges by the microbiota, and highlight important future directions and considerations for the use of antibiotics treatment in manipulation of the microbiota.

## Introduction

Over the past several decades, there has been a dramatic increase in both scientific and popular interest in the effects of the intestinal microbiota on human health. The microbiota, consisting of the bacteria, viruses, fungi, and archaea that inhabit different niches in the human body, has been implicated in regulation of inflammatory, infectious and metabolic diseases, and appears to play a critical role in potentially causing, propagating, or preventing human illnesses (Lai et al., 2014; Norman et al., 2014; Palm et al., 2015). With the surge of enthusiasm to understand this new and massively complex factor in human health has come the need to effectively model it. Specifically, the development of small animal models of the microbiota permits testing of subsets of the microbiota as causative vs. correlative factors in disease states, as well as offering a system to uncover putative therapeutics.

Two main methods have emerged to explore the effects of the microbiota on physiology and disease in mice: germ-free models and antibiotics treatment regimens. Both approaches have strengths and weaknesses. Here we will discuss commonly used regimens and methods to deplete the microbiota, the effects of these approaches on host physiology including cellular composition, signaling pathways, and organ function, and briefly describe what has been found using these two different methods to model the effects of the microbiota on human disease.

## Germ-free and antibiotics treatment models

Germ-free mice are bred in isolators which fully block exposure to microorganisms, with the intent of keeping them free of detectable bacteria, viruses, and eukaryotic microbes. Initially conceptualized by Louis Pasteur in 1885, colonies of germ-free rodents were first established in the 1940s (Yi and Li, 2012; Al-Asmakh and Zadjali, 2015). Germ-free mice allow for study of the complete absence of microbes or for the generation of gnotobiotic animals exclusively colonized by known microbes. However, generating and maintaining these mice requires specialized facilities, and the cost, labor, and skills required to maintain them can make these models inaccessible to many researchers. Germ-free mice must be monitored regularly for contamination using a combination of culturing, microscopy, serology, gross morphology, and sequencing-based detection techniques (Fontaine et al., 2015; Nicklas et al., 2015). For example, Charles River, one common germ-free vendor, routinely uses a serologic assay to test for viral contamination including murine norovirus, mouse rotavirus, and mouse cytomegalovirus; PCR (both 16S and pathogen-specific), microscopy, and culturing to test for bacteria; and gross examination of animals to test for parasites (Charles River Germ-Free Mouse Report). Additionally, any unique mouse strain to be studied under germ-free conditions must be rederived in these facilities, and this limits the number of different genotypes that are feasible to study. Further, maintenance of mice in isolators may make it impractical or challenging to conduct some studies (for example, behavioral testing or pathogen infections).

**Figure 1:**
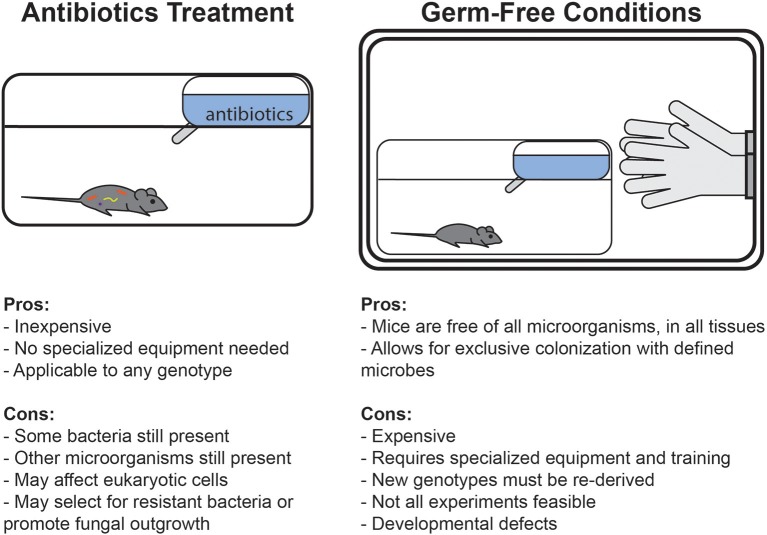
Comparison of the advantages and disadvantages of germ-free and antibiotics-treated mouse model systems.

An alternate method that has emerged to avoid some of these complications has been the use of antibiotics treatment (Figure 1). Treatment with broad-spectrum antibiotics is commonly used to deplete the gut microbiota of mice, and can be readily applied to any genotype or condition of mouse. Unlike germ-free conditions, under which complete sterility is maintained throughout life, antibiotics can deplete bacterial populations in mice which were normally colonized since birth. Germ-free animals are broadly impaired in many aspects of development and early immune education, whereas antibiotics treatment in adult mice specifically allows for study of the role of bacteria in maintaining cell functionality and signaling pathways after development. Alternatively, some studies deliver antibiotics in drinking water to pregnant dams to limit maternal transfer of microbes and then maintain the cage on the regimen during weaning to study the effects of bacterial depletion early in development (Lamousé-Smith et al., 2011; Deshmukh et al., 2014; Gonzalez-Perez et al., 2016; Li et al., 2017).

Due to differences in mechanism of action, antibiotics can selectively deplete different members of the microbiota. For example, metronidazole and clindamycin both target anaerobes, vancomycin is only effective against gram-positive bacteria, and polymyxin B specifically targets gram-negative bacteria (Atarashi et al., 2011; Schubert et al., 2015). Individual antibiotics can be used to shift the composition of the gut microbiota in order to identify classes of bacteria relevant to different phenotypes (Schubert et al., 2015; Zackular et al., 2016). In contrast, a cocktail of different classes of antibiotics can be used to broadly deplete the gut microbiota. Researchers have used various regimens which differ in antibiotic combination, dose, and length of treatment (Table 1). All of these combinations broadly target Gram-positive, Gram-negative, and anaerobic bacteria. Often, antibiotics are diluted in drinking water and mice are allowed to drink *ad libitum* throughout the course of treatment; therefore, actual delivered doses can vary. Some protocols additionally include antifungals in the cocktail to avoid fungal overgrowth during treatment (Reikvam et al., 2011; Grasa et al., 2015; Zákostelská et al., 2016). Many also add sweeteners such as sugar, Splenda, or Kool-aid to mask any bitterness and ensure mice drink the antibiotics-containing water (Abt et al., 2012; Baldridge et al., 2015; Emal et al., 2017). However, there are reports of mice avoiding water and becoming dehydrated when provided antibiotics in this manner (Hill et al., 2010; Reikvam et al., 2011; Zákostelská et al., 2016). Daily oral gavage can prevent dehydration and allow delivery of a precise dose of antibiotics, so this method is sometimes used alone or in combination with delivery in drinking water, though it is more labor-intensive (Kuss et al., 2011; Reikvam et al., 2011).

**Table 1:** Broad-spectrum antibiotics treatment regimens.

**Method**	**Antibiotics**	**Concentration**	**Duration**	**Additions**	**References**
Drinking water (*ad libitum*)	Vancomycin + metronidazole	0.5–1.0 g/L each	10 weeks		Atarashi et al., 2008
	Ciprofloxacin + metronidazole	1 g/L each	2 weeks		Josefsdottir et al., 2017
	Vancomycin + ampicillin + polymixin	0.1–1.0 g/L each	4 weeks		Kim et al., 2017
	Vancomycin + neomycin + metronidazole	0.5–1.0 g/L each	7 days		Brandl et al., 2008; Kinnebrew et al., 2010
			2 weeks	Kool-Aid	Josefsdottir et al., 2017
	Streptomycin + colistin + ampicillin	1–5 g/L each	6 weeks	2.5% sucrose	Sawa et al., 2011
	Ampicillin + neomycin + streptomycin + vancomycin	0.5–1.0 g/L each	4–5 weeks		Khosravi et al., 2014
	Cefoxitin + gentamicin + metronidazole + vancomycin	1 g/L	10 days		Ganal et al., 2012
	Gentamicin + ciprofloxacin + streptomycin + bacitracin	0.15–2 g/L each	4 weeks	3% sucrose	Yan et al., 2016
	Vancomycin + neomycin + kanamycin + metronidazole	0.5–1.0 g/L each	3 weeks		Gury-BenAri et al., 2016
	Vancomycin + ampicillin + kanamycin + metronidazole	0.5–1.0 g/L each			Levy et al., 2015
	Vancomycin + neomycin + ampicillin + metronidazole	0.35–1.0 g/L each	7 days	3% sucrose, 1% glucose, or Kool-aid	Ochoa-Repáraz et al., 2009
			2 weeks		Hägerbrand et al., 2015; Hashiguchi et al., 2015; Knoop et al., 2015; Brown et al., 2017; Emal et al., 2017; Josefsdottir et al., 2017; Steed et al., 2017; Burrello et al., 2018; Thackray et al., 2018
			3 or more weeks		Rakoff-Nahoum et al., 2004; Ivanov et al., 2008; Vaishnava et al., 2008; Ichinohe et al., 2011; Ismail et al., 2011; Yoshiya et al., 2011; Naik et al., 2012; Corbitt et al., 2013; Diehl et al., 2013; Balmer et al., 2014; Mortha et al., 2014; Oh et al., 2014; Johansson et al., 2015; Wu et al., 2015; Zhang et al., 2015; Park et al., 2016; Yan et al., 2016; Cervantes-Barragan et al., 2017; Ge et al., 2017; Li et al., 2017; Durand et al., 2018
			3 4-day treatments with 3 day rests		Adami et al., 2018
Gavage	Vancomycin + neomycin + ampicillin + metronidazole + gentamicin	200 μl of 0.5–1.0 g/L each by daily gavage	3 day		Kelly et al., 2015
			10 days		Hill et al., 2010
	Bacitracin + neomycin + streptomycin	200 mg/kg body weight	3 days		Sayin et al., 2013; Wichmann et al., 2013; Fernández-Santoscoy et al., 2015
	Neomycin + bacitracin	20 mg each in 200 μl by daily gavage	7 days	Pimaricin (anti-fungal), adjusted pH to 4	Grasa et al., 2015
Combination	Ampicillin by drinking water; vancomycin + neomycin + metronidazole by gavage	1.0g/L in water 10 ml/kg of 5–10 g/L by gavage every 12 h	10–21 days	Amphotericin B (anti-fungal)	Reikvam et al., 2011; Hintze et al., 2014
	Vancomycin + neomycin + ampicillin + metronidazole	10 mg each by daily gavage 0.5–1.0 g/L each in water	5 days gavage followed by 7–10 days drinking water		Kuss et al., 2011
	Kanamycin + gentamicin + colistin + metronidazole + vancomycin	200 μl of 0.35–4 mg/ml by daily gavage, and mixed 2:100 into drinking water	7 days gavage followed by administration in water		Bashir et al., 2004; Stefka et al., 2014
	Metronidazole + colistin + streptomycin by gavage, vancomycin by drinking water	0.3–2 mg each by daily gavage, and 0.25 mg/ml in water	2 weeks	Amphotericin B (anti-fungal)	Zákostelská et al., 2016
	Oral streptomycin + ampicillin in drinking water	20 mg/mouse orally and 1 g/L in drinking water	1–2 weeks		Kim et al., 2018
	Streptomycin by gavage, followed by vancomycin + neomycin + ampicillin + metronidazole by drinking water	100 mg/mouse for single gavage and 0.5–1.0 g/L in drinking water	single gavage followed by >7 days drinking water	1% sucrose	Kernbauer et al., 2014

Validation of bacterial depletion can be performed with culture-based methods by assessing the colony-forming units (CFUs) from fecal samples plated in aerobic and/or anaerobic conditions on non-selective media. However, this method only accounts for cultivatable microbes. Quantitative PCR of the gene encoding 16S rRNA allows for culture-independent assessment of gastrointestinal bacterial load. Broad-spectrum antibiotics treatment can decrease bacterial load by multiple orders of magnitude in 2 weeks of treatment or less (Baldridge et al., 2015; Gonzalez-Perez et al., 2016; Brown et al., 2017). Both germ-free and antibiotics-treated mice allow for the introduction of microbes in which the contributions of defined bacterial species or consortia can be studied (Tan et al., 2016; Staley et al., 2017). Although some phenotypes seen with antibiotics treatment are attributed to removal of a single bacterial species, many differences that occur are due to broad decreases in bacterial load. Consistent with this, treating bacterially-depleted mice with conserved pattern recognition receptor ligands such as flagellin (Ichinohe et al., 2011; Oh et al., 2014) or CpG dinucleotides (Ichinohe et al., 2011; Hill et al., 2012) can restore some defects, even in the absence of microbiota restoration.

Although most studies attribute phenotypic differences after antibiotics treatment to the depletion of gut microbes, some studies have assessed how regimens affect commensal populations at other sites. Oral antibiotic regimens can decrease culturable bacteria in the respiratory tract (Ichinohe et al., 2011; Abt et al., 2012; Brown et al., 2017) and the vagina of mice (Oh et al., 2016), but do not affect skin bacterial communities (Naik et al., 2012). Importantly, though rarely quantified, antibiotics treatment also likely drastically affects bacteriophage populations, though there is debate in the literature about whether phage play important roles in transfer of antibiotic resistance genes (Modi et al., 2013; Enault et al., 2017; Górska et al., 2018). Antibiotics treatment can allow for the outgrowth of commensal fungal species, potentially confounding results as these organisms can alter immune function, hence the inclusion of antifungals in some antibiotics treatment regimens (Noverr et al., 2004; Kim et al., 2014). An increasing appreciation for important roles for the virome and mycobiome may lead to enhanced interrogation of these effects in the future, as well as the potential impact of antibiotics on eukaryotic viruses and archaea (Norman et al., 2014). An important final potential disadvantage of antibiotics treatment can be the evolution or development of antibiotic-resistant bacteria and their subsequent selection and outgrowth in mouse intestines (Zhang et al., 2013; Morgun et al., 2015). Depending upon the starting bacterial composition, antibiotic cocktail, duration of treatment, and phenotypic read-out, antibiotic-resistance may confound findings in experiments, especially if resistant bacteria are present in only a subset of tested mice. Longitudinal analysis of bacterial populations in all experimental groups can aid in detection of resistance and analysis of whether resistant bacteria may affect experimental results.

Mice on antibiotics are not completely cleared of bacteria, but significant reductions in bacterial load are associated with shifts in cell populations, signaling pathways, and organ morphology, with results often paralleling what is seen in germ-free mice.

## Effects of the microbiota on cell populations and cytokines

**Figure 2:**
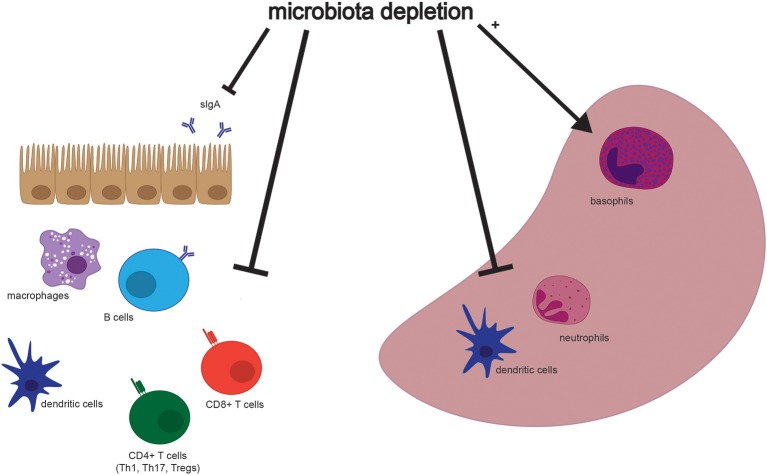
Selected effects of microbiota depletion on cells in the gastrointestinal tract and spleen. Populations of different cell types are altered in association with depletion of the microbiota in both the gastrointestinal tract **(Left)** and the spleen **(Right)**. Both secretory IgA (sIgA) and immune cell types are depleted in the intestine, while dendritic cells and neutrophils are depleted, but basophils are enriched, in the spleen. Please see Table 2 for more detailed findings by different groups and in other tissues.

Although many aspects of murine physiology are affected by microbial populations, the effects of antibiotics treatment on immune cell populations are some of the most well-studied (Figure 2). The immune system constantly responds to both pathogenic and commensal microbial populations, and shifts after antibiotics treatment reflect the dependence of cell populations and function on bacterial signals. While results of cell composition analysis are not uniform across studies, we will describe the most prominent and consistent observations (see Table 2 for exact findings by different groups).

**Table 2:** Effects of the microbiota on cell populations.

	**Broad-spectrum antibiotics**	**References**	**Germ-Free**	**References**
Monocytes	↓ in spleen similar in blood, BM ↓ inflammatory monocytes in BM, spleen, blood Similar inflammatory monocytes in vaginal mucosa after HSV-2 infection, in lung after flu infection	Abt et al., 2012; Balmer et al., 2014; Zhang et al., 2015; Oh et al., 2016; Josefsdottir et al., 2017	↓ or similar in blood ↓ in BM, spleen ↓ inflammatory monocytes in spleen, similar but trend ↓ in BM; similar in SI, colon Similar inflammatory monocytes in mesenteric LN after *S. typhimurium* infection	Balmer et al., 2014; Khosravi et al., 2014; Fernández-Santoscoy et al., 2015; Zhang et al., 2015; Hergott et al., 2016; Tan et al., 2016
	Increased inflammatory monocyte turnover and apoptosis in bloodstream; decreased migratory capacity of BM monocytes	Hergott et al., 2016; Emal et al., 2017	
Macrophages	↓ in SI, colon; ↓ or similar in spleen, liver; similar in PP, mesenteric LN, cervical LN, kidney, lungs; ↑ in BM Similar in lungs after *P. aeruginosa* or flu infection	Ochoa-Repáraz et al., 2009; Abt et al., 2012; Corbitt et al., 2013; Zhang et al., 2015; Ekmekciu et al., 2017; Emal et al., 2017; Robak et al., 2018	↓ or similar in spleen ↓ in liver	Ganal et al., 2012; Corbitt et al., 2013; Khosravi et al., 2014
	Less mature in kidney, liver, spleen; decreased cytokine production in lung after respiratory infection	Abt et al., 2012; Brown et al., 2017; Emal et al., 2017	
Dendritic cells (DCs)	↓ mDCs, pDCs in spleen; ↓ activated DCs in SI, colon, mesenteric LN, spleen; ↓ CD103+ DCs in lung; ↓ or similar CD103+ DCs in mesenteric LN Similar in lung/mediastinal LN after flu infection, similar in vaginal mucosa before or after HSV-2 infection	Abt et al., 2012; Hägerbrand et al., 2015; Oh et al., 2016; Ekmekciu et al., 2017; Thackray et al., 2018	↓ in spleen; ↓ or similar in mesenteric LN Similar in skin; similar cDCs in spleen	Walton et al., 2006; Naik et al., 2012; Hägerbrand et al., 2015
	Similar antigen-presentation abilities Similar surface markers in lung, altered in mesenteric LN, PP Impaired type 1 IFN production and priming of CD8 T cells after flu infection	Ochoa-Repáraz et al., 2009; Ichinohe et al., 2011; Abt et al., 2012; Ganal et al., 2012; Thackray et al., 2018	Decreased maturity but similar antigen presentation abilities impaired type 1 IFN production	Walton et al., 2006; Ganal et al., 2012
Granulocytes	↓ total in BM; similar in blood	Balmer et al., 2014; Josefsdottir et al., 2017	↓ total in BM; similar in blood	Balmer et al., 2014
Neutrophils	↓ in BM, spleen, blood; similar in liver, BALF, vaginal mucosa; ↑ in lung after *P. aeruginosa*, flu, *S. pneumoniae* or *K. pneumoniae* infection	Abt et al., 2012; Zhang et al., 2015; Hergott et al., 2016; Oh et al., 2016; Brown et al., 2017; Li et al., 2017; Robak et al., 2018	↓ or similar in spleen, BM ↓ blood similar in mesenteric LN after *S. typhimurium* infection ↓ in lung after *K. pneumoniae* infection	Fagundes et al., 2012; Khosravi et al., 2014; Fernández-Santoscoy et al., 2015; Sturge et al., 2015; Zhang et al., 2015; Hergott et al., 2016; Josefsdottir et al., 2017
	Decreased accumulation in BM or blood of neonates Increased turnover and apoptosis in bloodstream; Fewer aged neutrophils in blood	Deshmukh et al., 2014; Zhang et al., 2015; Hergott et al., 2016	Decreased accumulation in BM or blood of neonates Fewer aged neutrophils in blood	Deshmukh et al., 2014; Zhang et al., 2015
	Similar phagocytosis/reactive oxygen species production, adhesion in neonates; impaired neutrophil extracellular trap formation *in vitro*	Deshmukh et al., 2014; Zhang et al., 2015	
Basophils, eosinophils, mast cells	↑ basophils in blood, spleen Similar mast cells, eosinophils in blood ↑ eosinophils in inguinal subcutaneous adipose tissue, vaginal mucosa	Hill et al., 2012; Suárez-Zamorano et al., 2015; Oh et al., 2016	↑ basophils in blood, spleen similar eosinophils and mast cells in skin	Hill et al., 2012; Naik et al., 2012
	↑ eosinophils in lung/BALF after allergen exposure	Hill et al., 2012; Adami et al., 2018	
Lymphocytes	Similar in spleen; ↓ in lung, liver	Cheng et al., 2014	
αβ T cells	↓ in PP, mesenteric LN, cervical LN, SI, colon Similar or ↑ in BM Similar in blood, liver ↓ or ↑ in spleen	Ochoa-Repáraz et al., 2009; Zhang et al., 2015; Ekmekciu et al., 2017; Josefsdottir et al., 2017; Li et al., 2017	↓ in SI, blood, spleen Similar in skin ↑ in BM	Naik et al., 2012; Kernbauer et al., 2014; Zhang et al., 2015
CD4 T cells	↓ in PP, cervical LN, SI, colon, spleen, blood Similar in BM Similar or ↓ in mesenteric LN, SI Similar or ↓ or ↑ in spleen ↓ % CD4+ memory cells in SI, colon, mesenteric LN, spleen	Ivanov et al., 2008; Ochoa-Repáraz et al., 2009; Sawa et al., 2011; Kernbauer et al., 2014; Ekmekciu et al., 2017; Josefsdottir et al., 2017; Burrello et al., 2018; Thackray et al., 2018	↓ in SI, mesenteric LN, BM ↓ or similar in colon, spleen Similar, blood, cutaneous LN	Huang et al., 2005; Mazmanian et al., 2005; Atarashi et al., 2008; Sawa et al., 2011; Naik et al., 2012; Sjögren et al., 2012; Smith et al., 2013; Kernbauer et al., 2014
	Impaired activation after HSV-2 infection	Oh et al., 2016	
Th1 cells (Ifnγ+)	↓ in SI, colon Similar in mesenteric LN, spleen, vaginal mucosa or draining LNs, skin	Naik et al., 2012; Kernbauer et al., 2014; Oh et al., 2016; Ekmekciu et al., 2017	↓ in SI, mesenteric LN, colon, skin Similar or ↓ in mesenteric LN Similar in cecal patch, colon ↓ in draining lymph nodes after EAE induction?	Zaph et al., 2008; Lee et al., 2011; Naik et al., 2012; Kernbauer et al., 2014
	↓ IFNγ response to flu Similar IFNγ response to OVA, respiratory HSV-2, *L. pneumophila* ↑ IFNγ response to *Salmonella* in mesenteric LN, SI	Ichinohe et al., 2011; Diehl et al., 2013; Kim et al., 2018	
Th2 cells (IL4+)	↑ in mediastinal LN after allergen exposure	Hill et al., 2012	
Th17 cells (IL17+, Rorc+)	↓ in SI, colon, mesenteric LN, spleen Similar in skin, liver	Atarashi et al., 2008; Ivanov et al., 2008; Sawa et al., 2011; Naik et al., 2012; Ekmekciu et al., 2017; Li et al., 2017	↓ in colon, cecum, mesenteric LN, skin Similar or ↓ in PP Similar or ↓ or ↑ in SI ↑ in cecal patch, colon	Atarashi et al., 2008; Ivanov et al., 2008; Zaph et al., 2008; Sawa et al., 2011; Naik et al., 2012; Kernbauer et al., 2014; Tan et al., 2016
T regulatory cells (FoxP3+)	↓ in colon Similar or ↓ in SI, spleen, PP ↓ or ↑ in mesenteric LN Similar in BM, liver ↑ in cervical LN, lung	Ivanov et al., 2008; Ochoa-Repáraz et al., 2009; Ichinohe et al., 2011; Mortha et al., 2014; Ekmekciu et al., 2017; Josefsdottir et al., 2017; Li et al., 2017; Thackray et al., 2018	↓ in PP, colon Similar in spleen, mesenteric LN, peripheral LN, cutaneous LN, cecal patch, colon, blood, thymus ↑ in SI, skin; ↑ in draining LN and spleen after EAE induction ↓ Rorγt+ T_regs_ in colon, SI, MLN; similar or ↓ Helios+, similar Gata3+ T_regs_ in colon	Ivanov et al., 2008; Zaph et al., 2008; Lee et al., 2011; Naik et al., 2012; Smith et al., 2013; Ohnmacht et al., 2015; Durand et al., 2018
CD8+ T cells	↓ in SI, colon, blood Similar or ↑ in mesenteric LN ↓ or ↑ in spleen ↑ in PP, cervical LN, BM	Ochoa-Repáraz et al., 2009; Kernbauer et al., 2014; Ekmekciu et al., 2017; Josefsdottir et al., 2017; Thackray et al., 2018	↓ in mesenteric LN Similar in SI, colon, blood, cutaneous LN, spleen	Huang et al., 2005; Naik et al., 2012; Kernbauer et al., 2014
	Similar IFNγ+ in SI, vaginal mucosa or draining LNs Impaired response to flu, vaginal HSV-2; similar response to OVA, respiratory HSV-2, *L. pneumophila*	Kernbauer et al., 2014; Oh et al., 2016 Ichinohe et al., 2011,?; Abt et al., 2012; Oh et al., 2016	↓ IFNγ+ in SI, colon, mesenteric LN	Kernbauer et al., 2014
CD4+CD8aa+ cells	↓ in SI epithelium	Cervantes-Barragan et al., 2017	↓ in SI epithelium	Cervantes-Barragan et al., 2017
γδ T cells	Similar IL-17+ in SI ↓ IL-17+ in liver	Ivanov et al., 2008; Li et al., 2017	Similar in skin Similar or ↑ in SI ↓ IL-17+ in SI, skin, liver	Bandeira et al., 1990; Ivanov et al., 2008; Ismail et al., 2011; Naik et al., 2012; Li et al., 2017
	Less activated and more apoptotic in liver, ↓ production of antimicrobials in SI	Ismail et al., 2011; Li et al., 2017	↓ production of antimicrobials in SI, less activated in liver, diminished response to mucosal injury in colon	Ismail et al., 2009, 2011; Li et al., 2017
NK T cells	Similar or ↓ in spleen Similar in PP, cervical LN, mesenteric LN, liver ↑ in colon	Ochoa-Repáraz et al., 2009; Li et al., 2017; Burrello et al., 2018	↑ in colon	Kernbauer et al., 2014
B cells	↓ in SI, colon, PP Similar or ↓ in spleen, blood, BM Similar in mesenteric LN, cervical LN, liver	Ochoa-Repáraz et al., 2009; Yoshiya et al., 2011; Zhang et al., 2015; Ekmekciu et al., 2017; Josefsdottir et al., 2017; Li et al., 2017; Thackray et al., 2018	↓ in blood Similar in spleen ↑ in BM ↓ IgA, IgG production in SI	Kernbauer et al., 2014; Zhang et al., 2015
Antibodies	Similar IgM, IgG in BALF, IgG in serum, ↑ in serum after allergen exposure ↓ IgA in BALF, blood, feces ↑ IgE in serum at baseline, after allergen exposure	Atarashi et al., 2008; Hill et al., 2012; Oh et al., 2014; Stefka et al., 2014; Uchiyama et al., 2014; Adami et al., 2018; Lynn et al., 2018; Robak et al., 2018	Similar IgG in serum, ↑ after allergen exposure ↓ IgA in feces ↑ IgE in serum at baseline, after allergen exposure	Atarashi et al., 2008; Hill et al., 2012; Oh et al., 2014; Stefka et al., 2014
	↓ antigen-specific response to vaccines in neonates, not adults ↓ flu-specific IgG, IgA after infection, IgG early after flu vaccine ↑*Salmonella*-specific IgG in blood and IgA in feces ↑ rotavirus-specific IgA in serum, feces, only at later times after infection	Ichinohe et al., 2011; Lamousé-Smith et al., 2011; Abt et al., 2012; Diehl et al., 2013; Oh et al., 2014; Uchiyama et al., 2014; Li et al., 2017; Lynn et al., 2018	↓ Ova-specific IgG in response to Ova immunization at all ages tested ↓ flu-specific IgM in serum after infection, IgG early after flu vaccine ↑ rotavirus-specific IgA, IgG in serum, only at later time points	Lamousé-Smith et al., 2011; Abt et al., 2012; Oh et al., 2014; Uchiyama et al., 2014
Innate lymphoid cells (ILCs)	↓ ILC3s and ILC1s in PP ↑ ILC3s in terminal ileum PP Similar or ↑ ILC3s in SI LP ↑ ILC2 in vaginal mucosa ↓ GM-CSF^+^ ILC3s in colon ILC1 and ILC2 expression becomes more ILC3-like	Sawa et al., 2011; Mortha et al., 2014; Hashiguchi et al., 2015; Gury-BenAri et al., 2016; Oh et al., 2016; Kim et al., 2017	↑ ILC2s in SI; similar activation Similar or ↑ ILC3s in SI Similar ILC1 in SI	Sawa et al., 2011; Kernbauer et al., 2014; Gury-BenAri et al., 2016; Schneider et al., 2018
Natural killer (NK) cells	↓ in spleen Similar in PP, mesenteric LN, cervical LN, liver	Ochoa-Repáraz et al., 2009; Li et al., 2017	Similar in spleen	Ganal et al., 2012
	Impaired cytotoxicity and cytokine production in spleen	Ganal et al., 2012	Impaired cytotoxicity and cytokine production in spleen	Ganal et al., 2012

*BALF, bronchoalveolar lavage fluid; BM, bone marrow; EAE, experimental autoimmune encephalomyelitis; LN, lymph node; PP, Peyer's patch; SI, small intestine*.

## Myeloid cells

Innate immune cells lack antigen-specific receptors, responding instead to broadly conserved microbial patterns. As innate cells continuously interface with the microbial populations constituting the microbiota, sensing of these microbes via pattern recognition receptors is essential for maintenance of normal host physiology (Chu and Mazmanian, 2013; Fawkner-Corbett et al., 2017).

Myeloid cell populations, which include macrophages, monocytes, and granulocytes, are broadly decreased in systemic sites after antibiotics treatment, similar to what is seen in germ-free mice (Khosravi et al., 2014). Although monocytes are generally not diminished in the bone marrow or peripheral blood of mice receiving antibiotics (Zhang et al., 2015; Josefsdottir et al., 2017), these cells have a reduced migratory capacity consistent with their decreased presence in peripheral tissues (Zhang et al., 2015; Emal et al., 2017). In contrast, the effects of antibiotics treatment on inflammatory monocytes and macrophages are more variable, with some groups reporting decreases in blood, bone marrow, and peripheral tissues (Zhang et al., 2015; Hergott et al., 2016; Ekmekciu et al., 2017) and others reporting no significant differences at baseline or after infection (Abt et al., 2012; Oh et al., 2016; Brown et al., 2017; Robak et al., 2018). Even when cell numbers are similar, macrophages are often less mature after antibiotics treatment, impairing their responses to pathogens (Abt et al., 2012; Brown et al., 2017; Emal et al., 2017).

Bulk granulocytes decrease in the bone marrow of antibiotics-treated mice, though their numbers in peripheral blood are similar (Balmer et al., 2014; Josefsdottir et al., 2017). Neutrophils decrease in bone marrow and in peripheral sites, with an increased rate of apoptosis and decrease in aged neutrophils in the bloodstream after microbiota depletion (Deshmukh et al., 2014; Zhang et al., 2015; Hergott et al., 2016). However, neutrophil populations are not diminished at the site of infection after pathogen exposure in antibiotics-treated mice (Abt et al., 2012; Oh et al., 2016; Brown et al., 2017; Robak et al., 2018). In contrast to neutrophils, the proliferation of basophil precursors in the bone marrow is increased after antibiotics treatment, associated with increased basophils in the periphery and an enhanced response to allergen exposure (Hill et al., 2012). Similarly, eosinophils in various tissues are enhanced at baseline (Suárez-Zamorano et al., 2015; Oh et al., 2016), and in the lung after inhaled allergen exposure (Hill et al., 2012). These alterations in granulocyte populations are consistent with a shift from type 1 to type 2 immune responses after depletion of the commensal microbiota.

Various dendritic cell subsets are also reduced after antibiotics treatment at both mucosal and systemic sites (Ichinohe et al., 2011; Ekmekciu et al., 2017; Thackray et al., 2018), although these differences may not be apparent after infection (Abt et al., 2012; Oh et al., 2016). Differences in dendritic cell numbers have not been reported in germ-free mice, though impairment in priming has been seen (Walton et al., 2006; Ganal et al., 2012).

Reductions in innate immune cell number and function, characteristic of both germ-free mice and antibiotics-treated mice, may be explained by diminished cytokine and chemokine levels, which are necessary for normal cell recruitment, differentiation, and functionality (Mortha et al., 2014; Brown et al., 2017). Reductions in myeloid populations are likely not explained by decreases in progenitor populations: although antibiotic exposure beginning *in utero* can reduce postnatal granulocytosis (Deshmukh et al., 2014), treatment in adult mice does not reduce myeloid progenitor populations in the bone marrow (Josefsdottir et al., 2017; Thackray et al., 2018).

## Lymphoid cells

In contrast to what is seen with myeloid progenitors, common lymphoid progenitors are reduced in the bone marrow after microbiota depletion (Josefsdottir et al., 2017; Thackray et al., 2018), consistent with what is seen in some, but not all, germ-free models (Balmer et al., 2014; Iwamura et al., 2017). Total lymphocytes are similarly reduced in the peripheral blood after antibiotics treatment (Josefsdottir et al., 2017).

The effects of the microbiota in regulating differentiated T cell populations has been widely explored, but results found are somewhat variable. αβ T cells generally decrease in peripheral organs (Ochoa-Repáraz et al., 2009; Zhang et al., 2015; Ekmekciu et al., 2017), although not in the bone marrow or blood (Zhang et al., 2015; Josefsdottir et al., 2017). Similarly, many reports suggest that CD4+ T helper cells decrease in tissues (Ochoa-Repáraz et al., 2009; Kernbauer et al., 2014; Ekmekciu et al., 2017; Josefsdottir et al., 2017; Thackray et al., 2018), as do CD4+ T memory cells (Ekmekciu et al., 2017), although others see either no difference or increases in specific tissues (Ivanov et al., 2008; Sawa et al., 2011; Ekmekciu et al., 2017; Josefsdottir et al., 2017; Burrello et al., 2018). Th1 cells tend to decrease in the gastrointestinal tract (Naik et al., 2012; Kernbauer et al., 2014; Ekmekciu et al., 2017), but not in extra-intestinal tissues (Naik et al., 2012; Oh et al., 2016; Ekmekciu et al., 2017), whereas Th17 cells decrease in most tissues studied (Atarashi et al., 2008; Ivanov et al., 2008; Sawa et al., 2011; Naik et al., 2012; Ekmekciu et al., 2017). The effects of microbial depletion on Th2 cells are less well-studied, although they have been seen to increase in lymph nodes after allergen exposure (Hill et al., 2012). Results with regulatory T cells are inconsistent across studies, with some citing decreases in different tissues (Ochoa-Repáraz et al., 2009; Mortha et al., 2014; Ekmekciu et al., 2017; Thackray et al., 2018), others seeing similar numbers regardless of antibiotics treatment (Ivanov et al., 2008; Ichinohe et al., 2011; Josefsdottir et al., 2017; Li et al., 2017), and still others seeing increases in some sites (Ochoa-Repáraz et al., 2009; Ichinohe et al., 2011).

Similar to CD4+ T cells, cytotoxic CD8+ T cells generally decrease in the intestine after antibiotics, though results at other sites are more varied (Ochoa-Repáraz et al., 2009; Kernbauer et al., 2014; Ekmekciu et al., 2017; Josefsdottir et al., 2017; Thackray et al., 2018). Proinflammatory cytokine production from cytotoxic T cells is not diminished at baseline after antibiotics treatment, but has been reported to decrease in response to infection with some pathogens (Ichinohe et al., 2011; Abt et al., 2012; Kernbauer et al., 2014; Oh et al., 2016).

Reports of B cell population shifts are varied, with some groups seeing declines in the blood, bone marrow, and tissues after antibiotics treatment, and others noting similar numbers regardless of microbiota depletion (Ochoa-Repáraz et al., 2009; Yoshiya et al., 2011; Zhang et al., 2015; Ekmekciu et al., 2017; Josefsdottir et al., 2017; Li et al., 2017; Thackray et al., 2018). Likewise, shifts in antibody responses are inconsistent—in general, total IgG and IgM levels remain similar in different sites analyzed, but secretory and serum IgA levels tend to decrease and serum IgE levels increase after microbiota depletion (Hill et al., 2010; Oh et al., 2014; Uchiyama et al., 2014; Adami et al., 2018; Lynn et al., 2018; Robak et al., 2018). Antigen-specific response to infection or vaccination vary by pathogen, mouse age, and time point after exposure analyzed, but neonates in particular generally produce a less robust response to vaccination after exposure to antibiotics (Ichinohe et al., 2011; Lamousé-Smith et al., 2011; Abt et al., 2012; Diehl et al., 2013; Oh et al., 2014; Uchiyama et al., 2014; Li et al., 2017; Lynn et al., 2018).

Innate-like lymphocytes (including CD8αα+ T cells, γδ T cells, NK T cells) and innate lymphoid cells (ILCs, including natural killer cells) localize to barrier sites and are influenced by the presence of commensal microbes (Constantinides, 2018). Double-positive CD4+CD8αα+ T cells serve regulatory functions in the small intestinal epithelium and are diminished in antibiotics-treated mice, associated with a reduction in the bacterium *Lactobacillus reuteri* which induces this cell type (Cervantes-Barragan et al., 2017). γδ T cells are present in epithelial tissues, mediating tissue repair and monitoring microbial populations. Although studies in germ-free mice suggest that the microbiota is dispensable for these cells to home to the intestine or skin (Bandeira et al., 1990; Ismail et al., 2011; Naik et al., 2012), both germ-free and antibiotics-treated models indicate that microbial colonization is necessary for normal activation and production of antimicrobial compounds by these cells (Ivanov et al., 2008; Ismail et al., 2009, 2011; Naik et al., 2012; Li et al., 2017). Levels of NK T cells are generally similarly maintained in tissues after antibiotics treatment, though their activation has not been well-studied (Ochoa-Repáraz et al., 2009; Li et al., 2017; Burrello et al., 2018). Likewise, ILCs have not been extensively evaluated after antibiotics treatment, although multiple studies report shifts in the representation or function of ILC subsets at mucosal surfaces (Ochoa-Repáraz et al., 2009; Sawa et al., 2011; Ganal et al., 2012; Mortha et al., 2014; Hashiguchi et al., 2015; Gury-BenAri et al., 2016; Oh et al., 2016; Kim et al., 2017; Li et al., 2017).

## Cytokines

Although shifts in cytokine levels after antibiotics treatment are variable (Table 3), most studies that report differences describe a shift away from proinflammatory cytokines. Many associate microbiota depletion with decreases in the production of IL-1 family cytokines, Th1 cytokines such as IFNγ and TNFα, and IL-17 family cytokines. The production of these cytokines is often similar or reduced specifically in the gastrointestinal tract in naïve animals, but the diminished response becomes apparent after challenge with a pathogen, often at the site of infection. For example, although IL-1 family cytokine levels are similar, pro-IL-1 and pro-IL-18 are reduced in the vaginal mucosa and lung in antibiotics-treated mice at baseline, associated with a reduced production of IL-1 family cytokines in response to infection at each site (Ichinohe et al., 2011; Abt et al., 2012; Oh et al., 2016; Robak et al., 2018). Similarly, type 1 cytokines such as IFNγ and TNFα are generally present at similar levels in tissues after antibiotics treatment in uninfected mice but reduced at the site of infection in microbiota-depleted mice (Abt et al., 2012; Oh et al., 2016; Robak et al., 2018). Inflammatory Th17 cytokines such as IL-17 and IL-22 are generally reduced in the intestines even at baseline in antibiotics-treated mice (Hill et al., 2010; Deshmukh et al., 2014; Ekmekciu et al., 2017), whereas differences in tissues such as the lung become apparent after infection at that site (Deshmukh et al., 2014; Suárez-Zamorano et al., 2015). In parallel with this decrease in inflammatory cytokines, some reports suggest that there is an increase in the expression of Th2 family cytokines such as IL-4, IL-5, and IL-13, especially after allergen exposure, consistent with a shift from Th1 to Th2-type immunity after microbiota depletion (Hill et al., 2010; Suárez-Zamorano et al., 2015; Oh et al., 2016).

**Table 3:** Effects of the microbiota on cytokine signaling.

	**Broad-spectrum antibiotics**	**References**	**Germ-Free**	**References**
IL-1 family cytokines	Similar IL-1β in jejunum, colon, BM, BALF, liver; similar IL-1α in jejunum, BALF, liver ↓ pro-IL-1 in lung ↓ IL-1β, IL-1α in vaginal washes after HSV-2 infection; ↓ IL-1β in BALF after flu infection ↓ Pro-IL-18 in BALF, ↓ IL-18 in colon trend ↓ IL-18 in vaginal washes after HSV-2 infection ↑ IL-33 in vaginal mucosa	Ichinohe et al., 2011; Abt et al., 2012; Levy et al., 2015; Suárez-Zamorano et al., 2015; Oh et al., 2016; Yan et al., 2016; Li et al., 2017; Robak et al., 2018	↓ IL-1 in BM ↓ IL-1β in SI, colon, trend ↓ BM ↓ IL-1α in skin ↓ IL-18 in colon ↑ IL-33 in colon, SI	Naik et al., 2012; Shaw et al., 2012; Sjögren et al., 2012; Singh et al., 2014; Levy et al., 2015; Ohnmacht et al., 2015; Yan et al., 2016
Th1 cytokines	Similar IL-2 in liver ↓ IFNγ in SI, in vaginal mucosa after HSV-2 infection; similar IFNγ in SI, colon, vaginal mucosa at baseline ↓ or similar TNFα in colon, trend ↓ in BM, ↓ in lung after flu infection; similar TNFα in vaginal washes, SI, BALF ↓ IL-12 in spleen after LCMV-infection; similar IL-12 in colon, SI, vaginal washes with or without HSV-2 infection	Hill et al., 2010; Abt et al., 2012; Suárez-Zamorano et al., 2015; Oh et al., 2016; Yan et al., 2016; Ekmekciu et al., 2017; Li et al., 2017; Burrello et al., 2018; Robak et al., 2018	↓ TNFα in colon, BM, WAT; similar in popliteal LN; ↓ in lung after *K. pneumoniae* infection ↑ IL-12β, similar IL-12α in colon ↓ IFNγ, TNFα in skin, similar IFNγ in popliteal LN, spleen after *Leishmania* infection ↓ IFNγ in draining LN after EAE induction	Oliveira et al., 2005; Zaph et al., 2008; Lee et al., 2011; Caesar et al., 2012; Fagundes et al., 2012; Naik et al., 2012; Sjögren et al., 2012; Yan et al., 2016
Th2 cytokines	↑ IL-4 in inguinal subcutaneous adipose tissue, in mediastinal LN after allergen exposure; similar in SI, vaginal washes ↑ IL-5 in inguinal subcutaneous adipose tissue, vaginal mucosa; similar in SI Similar IL-6 in SI, BM, vaginal washes, BALF, liver; ↑ in BALF after *P. aerigunosa* infection; similar or ↓ in colon; ↓ in BALF after flu infection, in spleen after LCMV infection Similar IL-10 in spleen, lung; similar or ↓ in SI; ↓ in colon Similar IL-13 in SI; ↑ in inguinal subcutaneous adipose tissue, in mediastinal LN after allergen exposure	Rakoff-Nahoum et al., 2004; Abt et al., 2012; Hill et al., 2012; Suárez-Zamorano et al., 2015; Oh et al., 2016; Yan et al., 2016; Ekmekciu et al., 2017; Li et al., 2017; Burrello et al., 2018; Robak et al., 2018	Similar IL-6 in colon; similar or ↑ in BM; similar or ↓ in SI Similar IL-10 in colon; ↓ IL-10 in WAT Similar IL-13 in colon ↑ IL-10 in lung after *K. pneumoniae* infection Similar IL-4 in popliteal LN, spleen after *Leishmania* infection	Oliveira et al., 2005; Zaph et al., 2008; Caesar et al., 2012; Fagundes et al., 2012; Shaw et al., 2012; Sjögren et al., 2012; Ohnmacht et al., 2015; Yan et al., 2016
Th17 cytokines	↓ IL-22 in SI, colon Similar IL-17 in lung; similar or ↓ in SI, colon; ↓ in liver; ↓ in lung after *S. pneumoniae* or *K. pneumoniae* infection	Hill et al., 2010; Deshmukh et al., 2014; Suárez-Zamorano et al., 2015; Brown et al., 2017; Ekmekciu et al., 2017; Li et al., 2017; Burrello et al., 2018	↓ IL-17 in SI; ↑ IL-17 in colon ↓ IL-17 in draining LN after EAE induction	Ivanov et al., 2008; Zaph et al., 2008; Deshmukh et al., 2014

*BALF, bronchoalveolar lavage fluid; BM, bone marrow; EAE, experimental autoimmune encephalomyelitis; LN, lymph node; SI, small intestine; WAT, white adipose tissue*.

Broadly, there is reasonable concordance between germ-free and antibiotics treatment mouse models in alterations of cellular compartments and cytokines. However, different groups have reported disparate findings with different antibiotics treatment regimens, making it challenging to definitively categorize microbiota-mediated modulatory effects. We propose that distinct starting microbiota composition and distinct regimens likely underlie this variability, and highlight this as an area of much-needed standardization.

## Microbiota effects at the organ level

In addition to shifts in cell populations and signaling pathways, antibiotics treatment has been seen to affect organ morphology more broadly, both in the gastrointestinal tract as well as in extra-intestinal organs (Table 4). As the bulk of commensals reside in the gastrointestinal tract where they assist with digestion and interact closely with epithelial cells, it is not surprising that many changes are seen in intestinal physiology after microbial depletion. The length of the whole intestine or the colon is not affected, but the cecum becomes dramatically larger, transit time increases, and fecal pellet frequency and consistency can be altered (Grasa et al., 2015; Suárez-Zamorano et al., 2015; Ge et al., 2017). Moreover, villi become narrower (Kernbauer et al., 2014), cellular proliferation decreases (Reikvam et al., 2011; Ekmekciu et al., 2017), and features such as tuft cells (Wilen et al., 2018) or goblet-cell antigen passages (Knoop et al., 2015) are affected in specific regions of the gastrointestinal tract. Immune function in the intestines is also affected, as the production of antimicrobial peptides is reduced (Brandl et al., 2008; Vaishnava et al., 2008; Kinnebrew et al., 2010; Reikvam et al., 2011), Paneth cells granules are diminished (Kernbauer et al., 2014), Peyer's patches become less abundant and decrease in cellularity (Reikvam et al., 2011; Grasa et al., 2015; Hashiguchi et al., 2015), expression of Toll-like receptors is altered (Grasa et al., 2015), and tolerance to the commensal intestinal microbiota is impaired (Kim et al., 2018).

**Table 4:** Effects of the microbiota on individual organs.

	**Broad-spectrum antibiotics**	**References**	**Germ-Free**	**References**
Whole intestine	Similar length, ↑ transit time	Grasa et al., 2015; Ge et al., 2017		
Small intestine	↓ transit time similar apoptotic cells, fewer proliferating cells ↓ RegIIIγ and RegIIIβ production ↓ number of PP; ↓ cells in PP ↓ villus width, ↓ T cells/vilus ↓ granules/Paneth cell altered expression of TLRs similar tuft cells	Brandl et al., 2008; Vaishnava et al., 2008; Kinnebrew et al., 2010; Reikvam et al., 2011; Wichmann et al., 2013; Kernbauer et al., 2014; Grasa et al., 2015; Hashiguchi et al., 2015; Park et al., 2016; Ekmekciu et al., 2017; Durand et al., 2018; Schneider et al., 2018; Wilen et al., 2018	↓ transit time fewer proliferating cells ↓ RegIIIγ, RegIIIβ production ↓ villus width, ↓ T cells/vilus, ↓ cells in LP ↓ cells in PP ↓ mucus thickness, attachment to epithelium; mucus more attached ↓ granules/Paneth cell, ↓ lysozyme+ cells/crypt similar tuft cells ↑ bile acids	Vaishnava et al., 2008; Sayin et al., 2013; Wichmann et al., 2013; Kernbauer et al., 2014; Schütte et al., 2014; Johansson et al., 2015; Park et al., 2016; Durand et al., 2018; Schneider et al., 2018
Cecum	↑ size ↑ villus length and width ↓ SCFAs decreased thickness of muscularis propria	Hill et al., 2010; Corbitt et al., 2013; Kelly et al., 2015; Park et al., 2016; Yan et al., 2016	↑ size ↑ villus length and width ↓ SCFAs, bile acids Decreased thickness of muscularis propria	Hill et al., 2010; Corbitt et al., 2013; Sayin et al., 2013; Smith et al., 2013; Yan et al., 2016
Colon	Similar length, ↑ transit time ↓ RegIIIγ and RegIIIβ, other anti-microbial factors ↓ epithelial regeneration, ↓ proliferating cells Similar mucus penetrability Altered expression of TLRs ↓ tuft cells ↓ SCFAs Formation of goblet-cell antigen passages	Reikvam et al., 2011; Wichmann et al., 2013; Grasa et al., 2015; Johansson et al., 2015; Knoop et al., 2015; Ekmekciu et al., 2017; Ge et al., 2017; Wilen et al., 2018	↓ RELMβ, other anti-microbial factors ↓ crypt height Similar mucus thickness, attachment to epithelium; decreased impenetrable mucus Similar tuft cells ↓ SCFAs, bile acids Formation of goblet-cell antigen passages	He et al., 2003; Matsumoto et al., 2012; Sayin et al., 2013; Wichmann et al., 2013; Kernbauer et al., 2014; Johansson et al., 2015; Knoop et al., 2015; Levy et al., 2015; McKinley et al., 2017
Lymph nodes	Similar or ↓ cellularity ↓ size and cellularity after flu infection	Ichinohe et al., 2011; Durand et al., 2018	↓ or similar cellularity Altered structure	Bauer et al., 1963; Manolios et al., 1988; Kernbauer et al., 2014; Zhang et al., 2015; Durand et al., 2018
Spleen	Similar or ↓ weight ↓ cellularity, fewer leukocytes	Ochoa-Repáraz et al., 2009; Reikvam et al., 2011; Yoshiya et al., 2011; Grasa et al., 2015; Suárez-Zamorano et al., 2015; Zhang et al., 2015; Josefsdottir et al., 2017; Thackray et al., 2018	Similar cellularity, similar lymphocytes Altered structure	Bauer et al., 1963; Mazmanian et al., 2005; Zhang et al., 2015
Thymus	↓ weight	Josefsdottir et al., 2017	Similar cellularity	Nakajima et al., 2014
Liver	Similar or ↓ weight Impaired regeneration Altered bile acid production	Corbitt et al., 2013; Sayin et al., 2013; Zhang et al., 2014; Wu et al., 2015; Yan et al., 2016	Similar weight Impaired regeneration Altered bile acid production	Cornell et al., 1990; Corbitt et al., 2013; Sayin et al., 2013; Yan et al., 2016
Fat	↓ weight of abdominal fat pads ↓ inguinal and perigonadal adipose tissue	Suárez-Zamorano et al., 2015; Yan et al., 2016	↓ weight of abdominal fat pads ↓ % body fat	Caesar et al., 2012; Yan et al., 2016
Bone	↑ bone mass	Yan et al., 2016	↑ bone mass vs. short-term SPF colonized, ↓ bone mass/length vs. long-term SPF colonized ↑ bone mass vs. conventional	Sjögren et al., 2012; Yan et al., 2016

*PP, Peyer's patch; TLR, Toll-like receptor; LP, lamina propria; SCFA, short-chain fatty acid; SPF, specific-pathogen-free*.

Non-gastrointestinal organs also depend on ongoing bacterial signals to maintain normal morphology. The spleen (Ochoa-Repáraz et al., 2009; Reikvam et al., 2011; Yoshiya et al., 2011; Zhang et al., 2015; Josefsdottir et al., 2017; Thackray et al., 2018), thymus (Josefsdottir et al., 2017), and lymph nodes (Ichinohe et al., 2011; Durand et al., 2018) may decrease in size and/or cellularity after antibiotics treatment. Liver regeneration is impaired in antibiotics-treated mice (Wu et al., 2015) and bile acid synthesis is altered (Sayin et al., 2013; Zhang et al., 2014). Additionally, fat pads diminish and bone mass increases, consistent with a role for the microbiota in maintaining normal body composition (Suárez-Zamorano et al., 2015; Yan et al., 2016). There have been a number of intriguing studies recently exploring the role of the microbiota in regulating brain function and behavior via the gut-brain axis; this complex topic has been recently reviewed elsewhere (Cryan and Dinan, 2012; Liu and Zhu, 2018).

## Microbiota regulation of immune challenges

As might be expected, given the systemic and tissue-specific differences in immune function, antibiotics-treated mice are more susceptible to a variety of pathogens. For example, microbiota-depleted mice are more susceptible to bacterial pathogens such as vancomycin-resistant enterococcus, *Salmonella*, and *Clostridium difficile* in the gastrointestinal tract (Kinnebrew et al., 2010; Fernández-Santoscoy et al., 2015; Theriot et al., 2016), a variety of pneumonia-causing bacteria in the respiratory tract (Brown et al., 2017; Robak et al., 2018), and systemic *Escherichia coli* (Deshmukh et al., 2014). Additionally, after antibiotics treatment, mice are impaired in their response to vaginal HSV-2 (Oh et al., 2016), flaviviruses (Thackray et al., 2018), influenza (Ichinohe et al., 2011; Abt et al., 2012), and cutaneous *Leishmania* (Naik et al., 2012). However, microbiota-depleted mice actually become less susceptible to enteric viral pathogens including murine norovirus and poliovirus (Kuss et al., 2011; Uchiyama et al., 2014; Baldridge et al., 2015), possibly due to direct interactions between viral pathogens and enteric bacteria or due to loss of specific cell types required for viral infection. Antibiotics-treated mice are additionally impaired in their development of tolerance to food antigens (Bashir et al., 2004; Kim et al., 2018) and are more prone to allergic diseases (Hill et al., 2012; Adami et al., 2018).

Many of the effects after antibiotics treatment in mice are consistent with what is seen in germ-free models, suggesting that these are dependent on regular signals from the microbiota. However, it is important to note that antibiotics can have effects on eukaryotes independently of the microbiota, as treatment of germ-free mice with antibiotics can replicate some findings seen when treating normally colonized mice (Han et al., 2015; Gopinath et al., 2018). Replicating key findings in germ-free mice can help confirm that the differences seen after antibiotics treatment are indeed caused by microbial depletion.

## Considerations for the future

While antibiotics treatment offers an inexpensive and accessible alternative to germ-free models, results obtained using these regimens come with the caveats of potential off-target drug effects and incomplete or inconsistent ablation of microbes. Additionally, because so many groups use distinct treatment regimens and mouse microbial populations may be institution-specific, antibiotics studies are much more challenging to compare than germ-free mouse studies.

We suggest that some standardization of antibiotics treatment regimens would be helpful; for example, if a standard cocktail were employed to demonstrate an initial finding, this could be compared to other studies, and subsequent follow-up experiments could be done with modified cocktails as necessary. Additionally, we suggest that at least a limited assessment of the replicability of findings in antibiotics-treated mice and germ-free mice would be of high value for most studies, to rule in or out potential off-target drug effects or developmental differences between germ-free and standard pathogen-free mice that may be important for a phenotype. Finally, it will be critical for investigators to ensure that microbial loads are consistently monitored in both antibiotics treatment and germ-free models to identify any effects of contaminants or antibiotic-resistant microbes.

Ensuring that we are able to interpret the contribution of an individual study to the field of microbiota research will require careful planning and execution of these experiments on the part of investigators. As we continue to uncover additional health and disease states in which the microbiota plays a role, the use of these models will become increasingly common.

## Author contributions

EK and MB wrote and edited the manuscript. KK edited the manuscript.

### Conflict of interest statement

The authors declare that the research was conducted in the absence of any commercial or financial relationships that could be construed as a potential conflict of interest.
